# Effects of caffeine or RX821002 in rats with a neonatal ventral hippocampal lesion

**DOI:** 10.3389/fnbeh.2014.00015

**Published:** 2014-01-28

**Authors:** Guy Sandner, Marie-Josée Angst, Thierry Guiberteau, Blandine Guignard, Astrid Nehlig

**Affiliations:** ^1^Faculté de Médecine, Université de Strasbourg, U1114 INSERMStrasbourg, France; ^2^Faculté de Médecine, Université de Strasbourg, UMR 7237 CNRS/UdSStrasbourg, France; ^3^Faculté de Médecine, Université de Strasbourg, U663 INSERMStrasbourg, France

**Keywords:** animal model of schizophrenia, ventral hippocampal lesion, xanthine, noradrenaline, dopamine, therapy

## Abstract

Rats with a neonatal ventral hippocampal lesion (NVHL) are used to model schizophrenia. They show enhanced locomotion and difficulties in learning after puberty. Such behavioral modifications are strengthened by dopaminergic psychostimulant drugs, which is also relevant for schizophrenia because illustrating its dopaminergic facet. But it remains questionable that only dopaminergic drugs elicit such effects. The behavioral effects could simply represent a non specific arousal, in which case NVHL rats should also be hyper-responsive to other vigilance enhancing drugs. We administered an adenosine (caffeine) or an adrenaline receptor antagonist, (RX821002) at doses documented to modify alertness of rats, respectively 5 mg/kg and 1 mg/kg. Rats were selected prior to the experiments using magnetic resonance imaging (MRI). Each group contained typical and similar NVHL lesions. They were compared to sham lesioned rats. We evaluated locomotion in a new environment and the capacity to remember a visual or acoustic cue that announced the occurrence of food. Both caffeine and RX82100 enhanced locomotion in the novel environment, particularly in NVHL rats. But, RX82100 had a biphasic effect on locomotion, consisting of an initial reduction preceding the enhancement. It was independent of the lesion. Caffeine did not modify the learning performance of NVHL rats. But, RX821002 was found to facilitate learning. Patients tend to intake much more caffeine than healthy people, which has been interpreted as a means to counter some cognitive deficits. This idea was not validated with the present results. But adrenergic drugs could be helpful for attenuating some of their cognitive deficits.

## Introduction

Neonatal lesion of the ventral hippocampus (NVHL) in rats produces an animal model currently used to investigate the developmental hypothesis of schizophrenia (reviews in: Lipska, 2004; Tseng et al., 2009). Even if no major damage was found to the ventral hippocampus in most schizophrenic patients, the model is still relevant since it involves developmental changes, especially within the dopaminergic mesocorticolimbic system now considered central to the model and to the disease (Halim and Swerdlow, 2000). Lesioned animals’ sensitivity to dopaminergic agents is enhanced, a number of basic emotional reactions are modified, and cognitive abilities adversely affected (Tseng et al., 2009). The hypersensitivity to dopaminergic drugs was demonstrated by administering indirect dopaminergic drugs, such as amphetamine or cocaine (Lipska et al., 1993; Chambers and Taylor, 2004; Corda et al., 2006). These drugs, as well as apomorphine a direct DA agonist, enhanced locomotion (Macedo et al., 2008, 2010; François et al., 2009; Bychkov et al., 2011; Sandner et al., 2011, 2012) and elicited behavioral modifications in NVHL rats that had been also documented with the set of tests as used in the present study (Macedo et al., 2008, 2010; Sandner et al., 2011, 2012).

But, it remains questionable that only dopaminergic drugs elicit the here over reported effects. Indeed, the behavioral consequence of the neonatal lesion could consist of a non specific arousal, in which case NVHL rats should also be hyper-responsive to any vigilance enhancing drug. Therefore we tested two arousing drugs, for instance caffeine, the effect of which only partially involves brain dopaminergic systems (Cauli and Morelli, 2005), and RX821002, a drug that elicits noradrenaline release in the cortex by blocking alpha2-adrenoautoreceptors in the locus coeruleus (Fresquet et al., 2007). Increased cortical noradrenaline release produces arousal and increases vigilance (González and Aston-Jones, 2006; Laloux et al., 2007). If the disorders elicited by dopaminergic psychostimulant drugs in NVHL rats exclusively depended on the alteration of the mesolimbic dopaminergic system, other vigilance enhancing drugs would alter less, or not at all the behavioral characteristics of NVHL rats than dopaminergic agents. There is an additional reason for having considered a noradrenergic agent, namely the increasing interest in the contribution of noradrenergic neuronal systems in the behavioral modifications of NVHL rats (Bhardwaj et al., 2004).

Efforts to interpret the modifications of behavior are hampered by rats’ emotional state readily modified by psychoactive drugs (Shah and Treit, 2003; Degroot and Treit, 2004). Although we used the lowest efficient doses determined in previous experiments of the two research groups participating to this study (Nehlig and Boyet, 2000; Fresquet et al., 2007), that were assumed to have no effect on mood, the question about the contribution of altered mood state is still relevant insofar as fear attenuation was demonstrated even in untreated NVHL rats subjected to the classical Elevated Plus Maze Test for anxiety (Pellow et al., 1985; Pellow and File, 1986; Macedo et al., 2008; Sandner et al., 2011; Lecourtier et al., 2012). Therefore this test was added to the present study.

The NVHL lesions have to be performed at the end of the first week of life. Their extension can be assessed before adulthood using magnetic resonance imaging (MRI), namely before experiments are conducted (Angst et al., 2007; Macedo et al., 2008, 2010, 2012; Bertrand et al., 2010; Sandner et al., 2010, 2011, 2012). Selecting rats on the basis of the MRI picture of their lesion provides optimal reproducibility of the experiments. This allowed selecting triplets of rats before the experiments which should limit variability. A member of each triplet received either caffeine, or RX1002, or saline. We tested the effect of caffeine (5 mg/kg), of RX821002 (1 mg/kg) and the vehicle in a Locomotion Activity Test, followed after a 4 days rest period by an Associative Conditioning Test that lasted during the subsequent month. At the end of the testing period, after 1 week of rest, rats were submitted to the Elevated Plus Maze Test. We used the same rats in the three tests. The validity of this method had been previously verified in experiments that we published in the past few years, finding no interference from one test to the subsequent one (Angst et al., 2007; Macedo et al., 2008, 2010, 2012; Sandner et al., 2010, 2011, 2012).

## Materials and methods

### Animals

The methodology and protocols were approved by the French Regional Ethics Committee of Alsace (CREMEAS) under the reference AL/03/03/02/11. All the procedures were conducted in accordance with French legislation and EU Directive 2010/63/EU for animal experiments.

**Figure 1:**
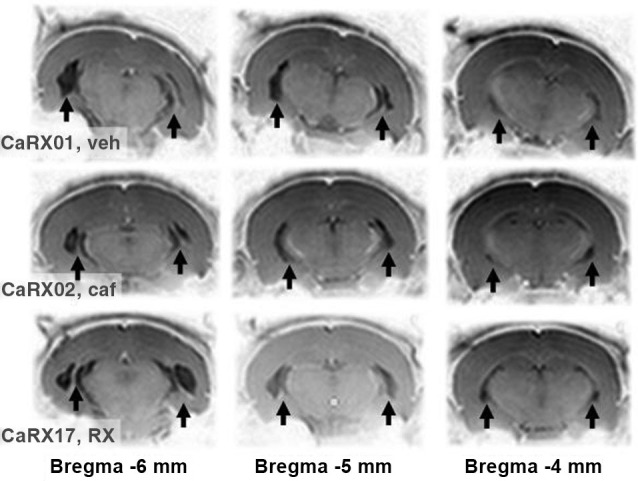
**Example of lesions according to MRI**. The contrasts of the pictures were inverted so that, opposite to the usual MRI images, the white matter and bone appear clear and the gray matter dark. With this inversion, the lesions replaced by cerebrospinal fluid appear black and the sclerosis, white. They are pointed by black arrows. The pictures in each row correspond to different coronal slices, the approximate anteriority relative to the bregma is indicated below. The three rows show an example of a triplet of lesions. Each row corresponds to one rat before it was treated either with vehicle, veh; or caffeine, caf; or RX821002. The identity of the rat was printed on the left most MRI picture (respectively CARX01,02, and 17) followed by the reference of its experimental group (veh, caf, and RX).

Twelve Sprague Dawley dam rats, each with 8 male pups, were purchased from Charles River (France), and housed on a 14/10 h light/dark cycle (lights on at 7 a.m.) with food and water provided *ad libitum*. When the pups were 7 days old, they were subjected to a lesion or sham lesion. MRI pictures of 4 week-old lesioned pups were then used to select those with bilateral lesions relevant for the experiments (see Figure 1). Behavioral tests were conducted in 10–20 week-old adult rats. Thereafter, the rats were killed, and histology was performed on brain sections to ascertain the exact extent of the lesion.

### Surgery

Surgical procedures were performed 7 days after birth under isoflurane anesthesia, as detailed previously (Angst et al., 2007). Either 0.3 µL of ibotenic acid (Sigma, France, 10 µg/µL, pH 7.4), in the case of NVHL rats (*N* = 62), or 0.3 µL of artificial cerebrospinal fluid, in the case of sham-lesioned rats (*N* = 34), was infused bilaterally into the ventral hippocampus with a 1 µL Hamilton syringe driven by a micro injection device (type 5000, David Kopf Instruments). The basic commercially available microinjection device was driven by a constant speed electrical motor. The tip of the injection needle was aimed at the ventral hippocampus (antero-posterior −3.0 mm, medio-lateral +3.5 mm, and ventro-dorsal −5.0 mm relative to bregma). After infusion, the needle was left immobile during 3 min. After both infusions, the skin was sutured and rats were allowed to recover on a heating pad before being returned to their dam (within 10 min after the end of the surgery). Three weeks after surgery, rat pups were weaned and housed two per cage.

### Selecting subjects using MRI imaging techniques

Twenty-one day-old lesioned pups were subjected to an MRI session under isoflurane anesthesia. MRI was performed on a small-animal scanner operating at 4.7 T (TR/TE/TEeff: 3000/30 ms/60 ms). A series of 10 slices (256 × 256 pixels) was generated over a 1 cm long section of the brain, rostral to the cerebellum-cerebrum gap, as in our previous studies and those conducted by others (Angst et al., 2007; Macedo et al., 2008, 2010, 2012; Bertrand et al., 2010; Sandner et al., 2010, 2011, 2012), the purpose being to select triplets of lesioned rats (1 saline, 1 caffeine and 1 RX821002), where each member of the triplet had about the same MRI image in terms of the location and symmetry of the lesion (examples are shown in Figure 1). We obtained 9 triplets of lesions (27 lesioned rats), to which we added 27 sham-lesioned controls. Rats that could not be included in a triplet were transferred to other research protocols.

Lesioned areas were drawn on MRI coronal sections. The numbers of pixels of the left and right lesions were summed up over successive rostro-caudal sections. The sum represents then the estimated volumes of the lesions. It was submitted to an ANOVA, with lesion side as within-group factor and treatment as between-group factor. Another ANOVA was computed on the sum of left and right lesions with the three rats in each triplet as within-group factor. The threshold for statistical significance for all statistical computations was set to *p* < 0.05.

### Treatments

A 3 × 2 experimental design was used (6 groups of 9 rats). The treatment was applied before each test and each learning session. The latency between injection and the beginning of the test was 10 min for caffeine (5 mg/kg) and 20 min for RX821002 (1 mg/kg), dissolved in saline (vehicle: veh) in a final volume of 1 ml and injected i.p. Control rats received a saline injection 10 or 20 min before testing. The following groups were considered: 9 NVHL rats treated with caffeine (caf group), 9 NVHL rats treated with RX821002 (RX group), and 9 NVHL rats which were given saline (veh group), plus three groups of 9 sham-lesioned rats which received the same treatments.

### Locomotion

Locomotion was evaluated in eight identical test cages (30 × 40 cm), which differed from the home cages in several regards: shape, dimmer light, silent environment, walls with black and white stripes. A passive infrared detector (IRP 124, Talco, France) was used to monitor activity during consecutive 5 min periods for 1 h. The detector comprised a Gallium-Arsenide infra-red sensitive surface placed behind a Fresnel array and reacted to the heat emitted by the rat. This array delimited 8 × 3 sectors. Any movement of the rat from one sector to another triggered an electric impulse sent to the computer interface. An ANOVA was computed to analyze the number of beam crossings. The 5 min periods were the within group factor (12 levels). The lesion (NVHL vs. sham) and treatment (caf vs. RX vs. veh) were the between group factors. As the situation changed significantly with time, the total number of beam crossings during the first 20 min of the test, and during the last 20 min, was subjected to two separate ANOVAs with the same between-group factors.

### Associative learning test

The rats were subjected to a conditioning procedure (Holland and Petrovich, 2005) which was adapted to characterize the cognitive effect of NVHL (Macedo et al., 2008). Three days before the procedure started they were deprived of food during the dark period. A recessed food dispenser was placed in one wall of an experimental chamber (25 × 35 cm) in the path of an infrared photo-beam to detect nose pokes. A light visual cue and loud speaker were placed above it. A microcomputer delivered cues or an edible reward (two 45 mg food pellets, Noyes rodent food pellets, Formula P, New Brunswick, USA) and recorded the breaks in the photo-beam. The rats were first trained to feed from the food dispenser. In this preparatory session, no cue was used, and the reward was given randomly five times in 30 min. The rats were then subjected to 8 days of two 30-min conditioning sessions, one in the morning and the other in the afternoon. In each session, there were five presentations of a 10 s cue, either light or a tone, immediately followed by the reward (Conditioned Stimulus (CS+ condition)). The rats were also subjected to a control condition consisting of five presentations of the other stimulus, with the food delivery system switched off (CS− condition). All the conditions were balanced: (1) for half of the rats, the CS+ was the tone, for the other half it was the light; (2) the morning/afternoon CS+/CS− sessions were spread randomly across the groups. The average nose-poke duration in the last 5 s of the 10 s cue was recorded as an index of the rats’ anticipation of the reward (Holland and Petrovich, 2005). The 5 s period preceding delivery of the CS served as a control. The total nose poke duration during these 5 s periods constituted the dependent variable of an ANOVA where these two periods (pre CS vs. end of CS) and the series of daily sessions were two within-group factors, and the lesion and treatment two between-group factors. We also performed ANOVAs on the mean nose-poke duration before delivery of the pellets during the first two daily sessions and the last two daily sessions, with lesion and drug as between-group factors. This allowed discriminating between learning stage and final performances of the rats (Angst et al., 2007; Macedo et al., 2008; Sandner et al., 2011, 2012).

### Anxiety, elevated plus maze test

The rats were subjected to the standard elevated Plus Maze Test for anxiety (Pellow et al., 1985; Pellow and File, 1986). The maze consisted of two wooden enclosed arms (50 cm long, 10 cm width, surrounded by 40 cm high walls), orthogonal to two open arms (same size as the enclosed walls, with a 2 cm high rim, 80 cm off the floor. The maze was placed in the same light environment and the same sound environment as the home cages. There were external cues around the maze. The rats were placed on a central platform facing one of the open arms. Their position was recorded for a period of 10 min. The time spent in the open and enclosed arms and the number of entries were subjected to ANOVAs, with the arms (open vs. enclosed) as within-group factor, and lesion and treatment as two between-group factors.

### Histology

The rats were given an overdose of pentobarbital (1 ml Doléthal, Vétoquinol, France). The rostral part of the brain, anterior to the occipital cortex-cerebellum junction, was immersed in 4% formalin and used to verify the lesion on 25 µm-thick sections stained with Cresyl violet.

## Results

### Lesioned brain areas

Photomicrographs representing MRI images of successive sections of a typical lesion are shown in Figure 1 for a triplet of rats. The triplet grouping strategy did not reduce the variability of the volume of brain damaged by the lesion (*F*_(2,16)_ = 1.48). The global ANOVA showed there was no significant difference either in lesion volumes on each side of the brain (*F*_(1,24)_ = 1.12) or in treatment groups (*F*_(2,24)_ = 0.15).

### Locomotion

**Figure 2:**
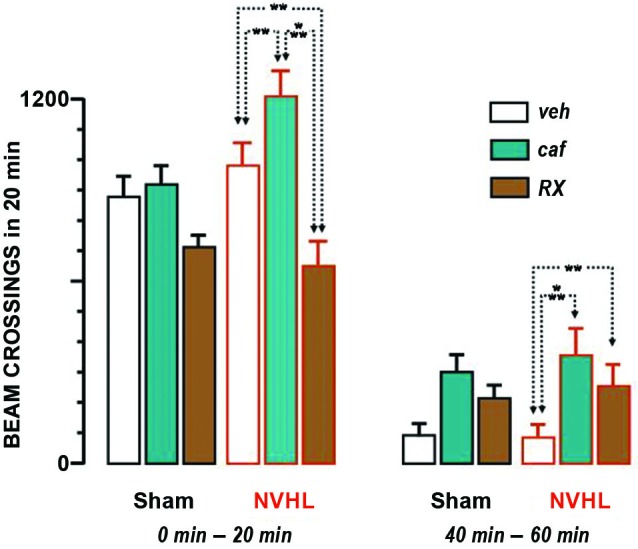
**Locomotion under drug or vehicle**. Bar graphs represent means + S.E.M. of the beam crossings during 20 min. The 6 bars on the left hand side represent the initial 20 min of the test. The 6 bars on the right hand side represent the last 20 min. In each group of 6 bars, the 3 left ones were obtained with sham-lesioned rats and the 3 right ones with NVHL rats (as indicated below the bars). The white bars represent the effect of the vehicle, the green one of caffeine and the brown one for RX821002. The dotted lines ending with two arrows indicate the differences between the groups that were statistically significant (** *p* < 0.01; *** *p* < 0.001).

An ANOVA performed with data pertaining to the whole testing period found a significant effect of treatment (*F*_(2,48)_ = 18.15, *p* < 0.0001) and time course (*F*_(11,528)_ = 83.44, *p* < 0.0001). There was no global effect of lesion (*F*_(1,48)_ = 2.13), and no interaction with the treatment (*F*_(2,48)_ = 2.61), although it was verging on significance (*p* ≃ 0.08). Given that the treatment interacted significantly with time (*F*_(22,528)_ = 3.92, *p* < 0.0001) we considered relevant to analyze the first 20 min separately. The effect of lesion remained marginal during this period (*F*_(1,48)_ = 3.19, *p* ≃ 0.08), but the effect of treatment was significant (*F*_(2,48)_ = 17.41, *p* < 0.001) and interacted significantly with the lesion (*F*_(2,48)_ = 5.33, *p* < 0.01). Fisher *post-hoc* tests revealed differences between all three treatment groups (caf-veh, *P* < 0.01; RX-veh, *p* < 0.01; caf-RX, *p* < 0.0001), as illustrated in Figure 2 caffeine enhanced locomotion and RX821002 reduced it. The picture for the last 20 min was different. The effect of lesion was not significant (*F*_(1,48)_ = 0.17) but the effect of treatment was (*F*_(2,48)_ = 6.40, *p* < 0.01), and it did not interact with the lesion (*F*_(2,48)_ = 0.12). Fisher *post-hoc* tests showed that the treatment groups differed significantly from vehicle (caf-veh, *P* < 0.001; RX-veh, *p* < 0.05), but not from each other (caf-Rx, NS). In the 40–60 min period locomotion was enhanced by both caffeine and RX821002.

In summary, caffeine enhanced locomotion throughout the test. Its effect interacted with the enhanced locomotion elicited by the lesion only for the first 20 min. RX821002 elicited a biphasic effect, consisting of a reduction and subsequent enhancement of locomotion, with both phases remaining independent of the effect of the lesion.

### Associative learning

**Figure 3:**
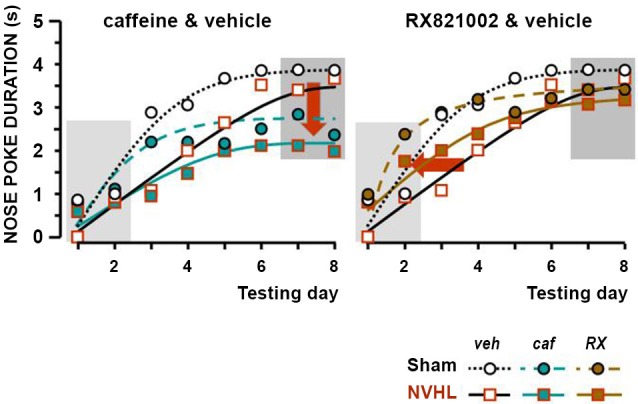
**Effect of the treatments in the associative learning test**. The two graphs show the duration of nose poking in the food magazine during the 5 s before food was delivered (ceiling = 5 s). The *X*-axis depicts the sequence of daily training sessions in which 10 s of the cue preceded food delivery. The *Y*-axis presents the mean nose poke duration for each daily session (1–8 days). Data of sham-lesioned rats were represented as circles and dotted lines, those of NVHL rats as squares and full lines. The white symbols correspond to the reference data (vehicle), the green symbols to the effect of caffeine, and the brown symbol to the effects of RX821002. The standard errors were not plotted for clarity (see statistical computations in the Result section). The dark backgrounds signal respectively the initial learning and final stable performances. The arrows point to the changes (left graph: effect of caffeine on the final performance and, right graph: effect of RX821002 on the learning stage).

The results of the rewarded associative learning experiments, expressed as nose-poke durations in the last 5 s of the conditioned stimulus (CS+), just before food delivery, are shown in Figure 3. For the sake of greater clarity, the effect of caffeine (left-hand graphs) is shown separately from the effect of RX821002 (right-hand graphs). Values for the CS− control group that stayed close to zero were not plotted. The two criteria for adequate learning were statistically met: there was a difference according to whether or not food was announced by the stimulus (CS+ vs. CS−: *F*_(1,48)_ = 262.42, *p* < 0.0001), as well as a progression—gradual learning was observed from session to session (*F*_(7,336)_ = 46.06, *p* < 0.0001). The effect of lesion was significant (*F*_(1,48)_ = 4.13, *p* < 0.05), but did not interact globally with treatment (*F*_(1,48)_ = 0.15). The interaction between treatment and learning chronology was significant (*F*_(7,336)_ = 3.08, *p* < 0.001). More associations were required in NVHL rats than their sham-lesioned controls (Figure 3). This was apparent in the vehicle control groups and reproduced results from our previous studies (Angst et al., 2007; Macedo et al., 2008; Sandner et al., 2011). The dynamics of the effect of caffeine differed from that of RX821002, as demonstrated by the extra statistical computation, where the learning stage (days 1 and 2) was considered separately from the final stage when most rats where quasi conditioned and therefore their performance was stable (days 7 and 8). In the learning stage, the lesion was not significant (*F*_(1,48)_ = 2.62), but treatment was (*F*_(2,48)_ = 3.88, *p* < 0.05), although it did not interact significantly with the lesion effect (*F*_(2,48)_ = 0.07). The *post-hoc* Fisher test revealed that the effect of RX821002 differed from that of vehicle (RX-veh, *p* < 0.05) and caffeine (RX-caf, *p* < 0.05; caf-veh, *NS*). RX821002 accelerated the learning rate in both sham-lesioned and NVHL rats, i.e., independently of the lesion effect. Once learning was established, the effect of the treatment became significant (*F*_(2,48)_ =6.65, *p* < 0.01), without interacting significantly with the lesion effect (*F*_(2,48)_ = 0.24). With RX821002, the final performance of NVHL rats was similar to that of sham-lesioned rats. The *post-hoc* Fisher test revealed that the effect of caffeine differed from that of vehicle (caf-veh, *p* < 0.001) and RX82002 (RX-caf, *p* < 0.05; RX-veh, *NS*). On the other hand, caffeine reduced the final performance levels of both sham-lesioned and NVHL rats.

### Elevated plus maze

**Figure 4:**
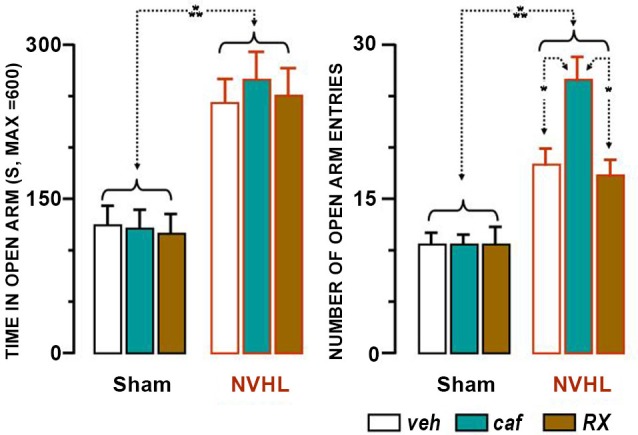
**Results of the Plus Maze Test**. Presence of the rats in open arms is shown in the left bar graph, and number of entries into the arms in the right bar graph. Each bar represents the mean + S.E.M. The groups of drugs and rats are similar to those described in Figures 2, 3. The white bars represent the effect of the vehicle, the green ones that of caffeine and the brown ones those for RX821002. The dotted lines ending with two arrows indicate the differences between the groups that were statistically significant (* *p* < 0.05; *** *p* < 0.001).

NVHL rats spent more time in the open arms and entered them more frequently than sham-lesioned rats (Figure 4). For the time spent in the open arms, the significant factor was lesion (*F*_(1,48)_ = 39.94, *p* < 0.0001). Treatment was not significant, neither *per se* (*F*_(2,48)_ = 0.08), nor in interaction with the lesion (*F*_(2,48)_ = 0.14). Lesion was also the significant factor for the number of entries (*F*_(1,48)_ = 35.84, *p* < 0.0001). Treatment was not significant, neither per se (*F*_(2,48)_ = 3.07, *p* ≃ 0.06), nor in interaction with the lesion (*F*_(2,48)_ = 2.99, *p* ≃ 0.06), but was verging on significance. *Post-hoc* statistical analyses provide the explanation for this marginal effect. The NVHL group of rats which were given caffeine entered the open arms more frequently than the other groups (caf-Veh, *p* < 0.05; caf-RX, *p* < 0.05; caf-veh, *NS*), and had a tendency to enter the enclosed arms more frequently than the RX group (caf-RX, *p* ≃ 0.06). The results illustrate the anxiolytic effect of the lesion, which persisted independently of both treatments and caffeine enhanced the number of entries both into the open and closed arms which may reflect increased locomotion.

### Histology

The ventral hippocampus appeared to be damaged exactly as observed with MRI. In most cases the lesion of the ventral hippocampus was only partial.

## Discussion

### Commented summary of the results

There was no significantly enhanced spontaneous locomotion in our NVHL rats, which does not mean that this did not exist. It deserves a brief comment because only a few experiments found no enhanced locomotion in NVHL rats (Wan et al., 1996). The increased locomotion reflects a single reaction to a complex situation where a diversity of factors have to be considered such as novelty, stress, animal housing and handling conditions, as well as many other experimental parameters (light and noise). It depended even on the strain and the sex of the rats (Lipska and Weinberger, 1995; Beninger et al., 2009). We shall not comment this point further because our aim was to analyze the reaction to drugs, not the baseline differences. But this aspect has to be kept in mind as the unusual drug-induced-state felt by the rat may trigger locomotion *per se* by its novelty or stressing properties (Lipska et al., 1993). Caffeine enhanced locomotion throughout the testing period, the most prominent enhancement occurring in the beginning when the environment was new to the rat. Caffeine did not modify the learning rate. The final performance of the rats was lower than in controls, meaning they spent less time with their snout in the food dispenser waiting for food once learning was acquired. Possibly the caffeine-elicited-fidgetiness could have prevented these rats from staying quietly with their snout in the food dispenser. Caffeine and RX821002, did not modify anxiety as assessed in a Plus Maze Test. RX821002 had a biphasic effect on locomotion, i.e., a reduction followed by enhancement, with both remaining independent of the neonatal lesion. Rats learned faster under RX821001 than under caffeine or vehicle. Each of these points will be commented in the next chapters of this discussion.

### Caffeine enhanced locomotion

Caffeine is a mixed antagonist of adenosine A1 and A2A receptors. At doses that enhance locomotion, adenosine A2 antagonists activate the same brain areas as dopaminergic agonists, as shown by *c-fos* activation and 2-deoxyglucose uptake studies (Svenningsson et al., 1997; Bennett and Semba, 1998; Nehlig et al., 1998; Nehlig and Boyet, 2000). These brain areas are anatomically and functionally modified in NVHL rats (Schroeder et al., 1999; Chrapusta et al., 2003; Corda et al., 2006; O’Donnell, 2010). This anatomo-functional convergence would easily account for the high sensitivity of NVHL rats to caffeine as well as the sensitization property that both drugs share (Simola et al., 2006; Ball and Poplawsky, 2011). Destroying dopaminergic neurons in the nucleus accumbens does not block caffeine-induced hyperlocomotion, which means that the neuronal networks involved in the action of caffeine or dopaminergic agents do not overlap (Swerdlow and Koob, 1985). Caffeine also activates midbrain–pontine areas involved in exploratory locomotion (Groenewegen and Trimble, 2007). It stimulates the locus coeruleus and its noradrenergic influence on neocortical neurons, therefore enhancing vigilance (Aston-Jones and Cohen, 2005) and, subsequently, locomotion (Svenningsson et al., 1997; Bennett and Semba, 1998; Nehlig et al., 1998; Nehlig and Boyet, 2000).

### Caffeine did not affect food-rewarded learning

Caffeine did not enhance the learning capacity of normal laboratory animals (Herz, 1999). Learning and memory improvements were reported only in exceptional cases, for instance when caffeine was administered after the learning stage (Angelucci et al., 1999, 2002). Caffeine did neither dramatically affect human performance in learning and memory tasks. Caffeine-enhanced alertness did not influence motor learning or verbal memory in healthy human subjects (Kelemen and Creeley, 2001; Mednick et al., 2008). But, learning seems to be facilitated in tasks where information was presented passively, and memory performance would appear to be improved somewhat under suboptimal alertness conditions (Nehlig, 2010). Some studies hinted at its capacity to reverse pathological memory failures, for instance in rats whose brains had been damaged (Gevaerd et al., 2001). This was not the case for NVHL rats in the learning test used for present study.

### Caffeine did not modify the level of anxiety (or fear)

During the initial researches using the Elevated Plus Maze Test, caffeine showed anxiogenic properties, but very high doses were administered (Pellow et al., 1985). All studies reporting anxiogenesis used doses of caffeine above 15 mg/kg in rats (Kayir and Uzbay, 2006; Park et al., 2010). Anxiogenic effects were also reported in humans, also at high doses (Jain et al., 2005). But, paradoxically, when yohimbine, that is anxiogenic in the Elevated Plus Maze Test, and caffeine were administered together, caffeine attenuated the anxiogenic effect of yohimbine (Baldwin et al., 1989). Caffeine also attenuated the anxiogenic effect occurring after benzodiazepine withdrawal (Baldwin et al., 1989). Thus, depending on the dose and experimental conditions, caffeine could be anxiogenic or anxiolytic. In the present experiments, the time spent in the open arms of the standard Elevated Plus Maze Test was not affected by caffeine. Furthermore, the anxiolytic effect produced by the neonatal lesion (Wood et al., 1997; Macedo et al., 2008; Beninger et al., 2009; Sandner et al., 2011; Lecourtier et al., 2012) was not sensitive to the effect of caffeine. Nevertheless, the number of open arm entries were enhanced by caffeine in NVHL rats. But this is usually seen as an expression of enhanced activity rather than anxiolysis (Lister, 1987; Silveira et al., 1993; Bhattacharya et al., 1997; Garcia et al., 2011).

### RX821002, an alpha-2 noradrenergic antagonist, modified locomotion

Inhibition of alpha-2-autoreceptors on cell bodies and dendrites, for example by administering RX821002, elicits release of noradrenaline in the prefrontal cortex (Florin et al., 1994; Fresquet et al., 2007). Infusing yohimbine, another alpha-2-adrenoceptor antagonist, into the locus coeruleus where are located the noradrenergic neurons innervating the cortex, reduced locomotion (Weiss et al., 1986). This is what we observed at the beginning of the locomotion test. But, Darracq et al. (1998) suggested that alpha-2-autoreceptor antagonists also facilitate dopaminergic transmission in the nucleus accumbens via a glutamatergic pathway running from the cortex to the ventral tegmental area (Darracq et al., 1998). The resulting secondary release of dopamine in the prefrontal cortex would then stimulate locomotion. This might explain why hyper-locomotion is delayed after RX821002. However, the dopaminergic neuronal circuits being modified in NVHL rats, this secondary hyper-locomotion should be different in NVHL rats compared to sham-lesioned rats, which was not the case in the present study (Flores et al., 1996, 2005; O’Donnell, 2010).

### An alpha-2-adrenoreceptor antagonist accelerated learning

The major finding of this study was that fewer learning sessions were needed when the alpha-2-adrenoreceptor antagonist, RX821002, was administered. The subsequent enhancement of learning performance partially compensated for the effect of the NVHL lesions. This is coherent with the known effects of another alpha-2-adrenoreceptor antagonist, atipamizole, which alleviated the deficits in mice whose noradrenergic system had been damaged as a result of the administration of DSP4 in the Morris Water Maze Learning Test (Björklund et al., 2000). Noradrenergic neurotransmission regulates many aspects of cognition, including working memory, arousal and attention (Aston-Jones and Bloom, 1981; Sirviö et al., 1992; Kubiak et al., 1998; Sirviö and MacDonald, 1999; Bhardwaj et al., 2004; Aston-Jones and Cohen, 2005). Under conditions of heightened arousal, such as the response to novelty or behaviorally significant stimuli, locus coeruleus noradrenergic neurons modulate functioning of the forebrain structures, such as the hippocampus, thalamus and cortex mainly, through postsynaptic adrenoreceptors (de Sarro et al., 1987; McCormick et al., 1991; Björklund et al., 2000). It will be interesting to further document the contribution of noradrenaline to the cognitive deficits of the NVHL model and in patients (Bhardwaj et al., 2004).

### The alpha-2-adrenoreceptor antagonist did not modify the level of anxiety (or fear)

RX821002 did not show any effect in the Elevated Plus Maze Test, either in sham-lesioned or in NVHL rats. This finding contrasts with observations of others, but obtained through an alternate experimental approach. For instance, anxiety assessed by the Elevated Plus Maze Test, was decreased after reduction of the expression of alpha-2A-adrenoreceptors in neonatal animals by means of anti-sense and RNA interference techniques (Shishkina et al., 2004).

### Interpretation of the contrasting effects of the two vigilance enhancing drugs used

Caffeine enhanced locomotion, a property common to dopaminergic drugs. Given that the other drug, RX821001, did not show this effect in NVHL rats shows that strengthening the hyper-locomotion of NVHL rats depends strictly on an hypersensitivity of brain dopaminergic systems. This reflects this standard point of view portrayed in the literature (reviews: Lipska, 2004; Tseng et al., 2009). In the learning test, RX821002 partially reversed the deleterious effect of the neonatal lesions, but caffeine did not. This indicates that the impaired prefrontal noradrenergic innervation of NVHL rats may contribute to the cognitive disruptions elicited by the neonatal lesion (Bhardwaj et al., 2004).

### Clinical relevance of the use of adenosine A2 receptor and adrenaline alpha-2-adrenoreceptor antagonists

Increased or excessive coffee intake among patients with schizophrenia is well documented (Lucas et al., 1990; Gurpegui et al., 2006; Strassnig et al., 2006). Patients are believed liking coffee because of the arousing effect of caffeine which may help to overcome the cognitive disruptions or apathy caused by the disease or by its medication. However, caffeine has been also reported to cause psychosis de novo (Shaul et al., 1984) and to exacerbate the symptoms of schizophrenia (Mikkelsen, 1978; Lucas et al., 1990).

Contrasting with the lack of improvement in the performance of rats under caffeine, RX821002, the alpha2-adrenoreceptor antagonist, improved learning. Research about the contribution of noradrenergic systems to schizophrenia has yielded inconsistent results (van Kammen and Antelman, 1984; van Kammen and Kelley, 1991; Yamamoto et al., 1994; Friedman et al., 1999; Klimek et al., 1999). Interest has been shown, however, in the prefrontal noradrenergic mechanisms and the potential role of alpha-2-adrenoreceptor antagonism in the antipsychotic effects of atypical neuroleptics, particularly considering that co-medication of fluphenazine with the alpha-2-adrenoreceptor antagonist idazoxan enhanced its antipsychotic and cognitive effectiveness (Litman et al., 1996). Our results complement these observations, highlighting the importance of adrenoreceptors as targets for treating cognitive difficulties like those experienced by patients with schizophrenia (McAllister, 2001; Masana et al., 2011).

## Author contributions

Guy Sandner scheduled the experiments. They provide answers to questions raised during a debate with Dr. Srivastava, from McGill University in Montreal, and Dr. Nehlig, from INSERM and the University of Strasbourg. Guy Sandner also analyzed the data and wrote the successive drafts of the manuscript, Marie-Josée Angst performed the lesions and conducted the experiments, Blandine Guignard and Thierry Guiberteau checked the extent of the lesions with MRI, Astrid Nehlig was involved in selecting the dose of drugs and testing methods. She also contributed to the manuscript.

## Conflict of interest statement

The authors declare that the research was conducted in the absence of any commercial or financial relationships that could be construed as a potential conflict of interest.
